# Implicit association tests for all: Using iatgen for non-English and offline samples

**DOI:** 10.1371/journal.pone.0342742

**Published:** 2026-04-17

**Authors:** João O. Santos, Emerson Do Bú, Tomohiro Hara, Cristina Mendonça, Sara Hagá, Ruth Pogacar, Michal Kouril

**Affiliations:** 1 CICPSI, Faculdade de Psicologia, Universidade de Lisboa, Lisbon, Portugal; 2 Institute of Social Sciences, University of Lisbon, Lisbon, Portugal; 3 Musashi University, Tokyo, Japan; 4 University of Calgary, Calgary, Alberta, Canada; 5 University of Cincinnati College of Medicine, Cincinnati, Ohio, United States of America; 6 Cincinnati Children’s Hospital Medical Center, Cincinnati, Ohio, United States of America; PLOS: Public Library of Science, UNITED KINGDOM OF GREAT BRITAIN AND NORTHERN IRELAND

## Abstract

The Implicit Association Test (IAT) has become an invaluable tool for researchers in many fields. The IAT is a sorting task that measures the strength of automatic associations between targets (e.g., flowers / insects) and attributes (e.g., pleasant / unpleasant). Several programs exist to create and run IAT studies, and each has unique advantages and disadvantages. Yet most share the same limitations: being general-purpose data collection tools that require time to master, requiring extra steps to run online (e.g., deploying a web server), and having no IAT-data analysis features. This increases researcher reliance on pre-made templates that typically operate only in English and are difficult to translate. Iatgen addresses some of these issues by allowing researchers to design and analyze IATs through a simple web-interface, to easily combine IATs with experimental manipulations or other measures in Qualtrics, and to analyze data using the same web-interface. However, until recently, the problem of monolingual, English-only capability remained. In this paper, we introduce iatgen’s new translation functionality, which allows users to create non-English IATs using the web-based iatgen Shiny app and the tr.iatgen R package. Users are invited to contribute to the translation repository in GitHub by submitting and reviewing IAT translations. We also describe a method for deploying Qualtrics-based IATs in offline environments. We hope this increased functionality will facilitate cross-cultural research and reduce the negative effects of disproportionately Western, educated, industrialized, rich, and democratic (WEIRD) samples.

## Introduction

The Implicit Association Test (IAT) [1] is one of the most popular tools for research in social cognition [2,3]. IATs are also used in economics [4], sociology and anthropology [5], marketing [6], medicine [7], and other fields. Many programs can be used to create and run IATs (e.g., Inquisit, OpenSesame, E-Prime). In the present paper, we discuss new language translations and offline functionality in one of those programs: iatgen.

### Using iatgen to reach Non-WEIRD samples

Iatgen [8] is a free, user-friendly software that allows researchers to design, conduct, and analyze IATs through a web-based interface and/or in R. Iatgen works in conjunction with Qualtrics (a data collection platform) and has the benefit of simplifying IAT set-up and data analysis based on established best practices [1,9–12]. Iatgen allows researchers to conduct IAT studies online (e.g., outside the lab), which makes data collection easier, faster, and possible for larger and more diverse samples. Indeed, recruiting participants for an iatgen-generated IAT via online platforms (e.g., Prolific) is as straightforward as recruiting for any Qualtrics survey—participants simply click a link, with no software download required. Moreover, iatgen simplifies the implementation of experimental manipulations and measures before or after an IAT. Finally, analyzing IAT data from an iatgen-generated experiment is as simple as uploading it to iatgen’s web interface for analysis (or loading the data into R and feeding it to the functions from the iatgen R package).

However, as with most research tools, iatgen is English-focused. For example, until recently, researchers using iatgen could easily include words in any language as stimuli, but IAT instructions (e.g., “Press E or I to advance to the next word/image”) were only available in English. Researchers working with non-English speaking samples went as far as manually translating iatgen source code [13] which is a time-consuming and error-prone task. Moreover, the existing documentation assumes that participation occurs in an internet-connected environment. These two features may have the unintended consequence of contributing to the disproportionate collection of Western, educated, industrialized, rich, and democratic (WEIRD) samples [14]. Such biased sampling may not be representative of the global population and has potentially negative consequences for scientific understanding. A review of comparative social and behavioral science studies found that WEIRD participants represent as much as 80% of those included in scientific research, but only 12% of the world’s population [15]. This presents a problem for generalizability. For instance, even fundamental processes like visual attention differ across cultures [16].

Here, we present new translation features to facilitate non-English IAT research, as well as instructions for offline data collection to increase researchers’ ability to conduct studies in environments without an internet connection. Iatgen is not the only software allowing researchers to program IAT studies, and there are several proprietary and open-source alternatives, namely MinnoJS (Implicit) in Qualtrics [17,18], Inquisit 6.0 [19], E-Prime 3 [20], and OpenSesame 3.2.8 [6,21] (see Table 1). However, iatgen may be the best option for researchers wishing to avoid WEIRD samples by providing multi-language data collection capabilities both online and offline.

**Table 1:** Comparisons between most commonly used software for IAT implementation.

Software	Paid	Data collection	Installation required	IAT analysis app	IAT language translation feature
**iatgen in Qualtrics**	Qualtrics subscription	Offline and Online^a^	No install required for online. Offline app available^a^	Yes	Yes
**MinnoJS (Implicit) in Qualtrics**	Qualtrics subscription	Offline and Online^a^	No install required for online. Offline app available	No	No
**Inquisit**	Yes	Offline and Online^b^	Yes	Yes	No^c^
**E-Prime**	Yes	Offline and Online^b^	Yes	No^d^	No
**OpenSesame**	No	Offline and Online^e^	Yes	No^d^	No

^a^ Qualtrics requires participants to install an app for offline data collection.

^b^ Requires participants to install an app or complete a download for online data collection.

^c^ Some multilanguage IAT templates exist, but there is no instruction translation feature.

^d^ Some templates for creating IATs in this software exist and may be provided with a separate data analysis script, but the feature is not built in.

^e^ Online data collection with OpenSesame requires it to be set up on a web server.

### The implicit association test

Greenwald and Banaji proposed that people’s behavior could be influenced by factors outside of conscious awareness (i.e., implicit factors), such as implicit self-esteem, attitudes, and stereotypes [22]. According to their theorizing, implicit cognition resulted from past experiences that might not be introspected (and thus might not be measurable through self-report), but could nonetheless influence people’s cognition and behavior. Greenwald and Banaji posited that the concept of implicit cognition could explain many effects, such as racial priming (in which presenting a racial prime influences subsequent interpretation of ambiguous stimuli), subliminal attitudinal conditioning (in which stimuli that are not consciously processed can impact attitudes), and mere ownership effects (in which the mere association of the self with an object leads to improved attitudes towards the object). The authors also called for the development of individual-level measures of this implicit cognition and noted that the two main avenues for such measurement development were reaction times and interpretation of ambiguous stimuli (i.e., projective measures). Later, Greenwald and collaborators developed the Implicit Association Test (IAT) [1] with the aim of measuring implicit attitudes by comparing the relative speed of physically associating certain targets (e.g., insects / flowers) with certain attributes (e.g., pleasant / unpleasant).

The IAT is designed to measure the extent to which the mental representations of a dichotomous target category (e.g., flowers / insects) and a dichotomous attribute category (e.g., pleasant / unpleasant) overlap. Test takers must categorize stimuli combinations as quickly and accurately as possible resulting in a reaction time measure. The IAT-specific statistic, called the *D* score, results from comparing the reaction times of different targets and attributes (e.g., flowers / unpleasant + insects / pleasant vs. flowers / pleasant + insects / unpleasant) [9]. Drawing on Greenwald and collaborators’ [1] original work, scholars developed many variations of the IAT tailored to specific purposes, such as the brief IAT [23], the pen-and-paper IAT [24,25], the pre-school IAT [26,27], and the single-category IAT [28].

Initially, the IAT displayed large variations in reliability (e.g., split-half correlations of .33 to .77 [9,31]), partially depending on scoring and methodological choices. The development of recommendations on scoring [9] and aspects such as the number of stimuli per category and the number of practice trials [31] resulted in improvements, with the IAT faring better than other implicit measures (with reliability varying from .70 to .93 vs. alternatives such as the Sorting Paired-Features task, the Affect Misattribution Procedure, and the Evaluative-Priming task which sometimes had reliabilities below .60 [29]). Still, recent research continues to find limitations in the psychometric qualities of the IAT (e.g., [30] in 2023 found split-half correlations that varied from .57 to .74), which have been at the heart of its continued theoretical (e.g., how stable across contexts and time should implicit bias be [32]) and methodological refinements (e.g., how to best capture stable traits using IATs [30]).

Beyond the psychometric issues, researchers have also raised other issues with the IAT. For instance, researchers have found that IAT results can be deliberately faked, at least when participants are instructed to [33]. IATs are also affected by the context of administration, such as whether the context is public or private [34]. Moreover, IATs are influenced by stable non-attitudinal/stereotypical factors, such as the cognitive capacity to switch mental sets [35] (for an in-depth discussion of IAT-related issues see [33,36,37]).

However, beyond these questions on how well and what the IAT measures, a strong argument in favor of its continued use is its ability to predict variance in judgment and behavior beyond self-reported attitudes and stereotypes, particularly in terms of interracial and intergroup behavior [38] (but see [39] for a critical reanalysis of data supporting IAT’s discriminant validity and [40] for a response to that critical reanalysis). While a single IAT should not be used to diagnose individuals (e.g., [30,33]), especially given the issues with reliability and the influence of extraneous factors, the IAT can be a useful tool in many research paradigms, such as those that assess experimental interventions and control for plausible influences the intervention may have on extraneous factors, such as faking [33] (see also [30,37,41] for further discussion).

Thus, while imperfect, the IAT remains an important research tool. The software in focus in the current paper, iatgen, provides two main benefits. First, iatgen can increase IAT’s flexibility, by facilitating both online and offline functionality in multiple languages. Second, it may attenuate some of the aforementioned issues. For instance, although IAT results may be deliberately faked, it is difficult for participants to misrepresent their true preferences while following the task instructions [3]. Attempted false responses, such as deliberately slow button pressing, are often excluded by the default data cleaning procedures in iatgen [3]. Furthermore, iatgen may improve data quality for studies that are likely to be affected by public administration (e.g., socially sensitive topics), as it allows participants to take IATs in their own homes. Finally, iatgen’s consistent application of the proper scoring guidelines reduces the impact of extraneous variables (e.g., the impact of central executive functions is decreased when following proper guidelines [35]). The purpose of this paper is to bring iatgen’s streamlining of the process of setting up and analysing IAT studies to non-English and offline uses.

## Materials and methods

The protocol described in this peer-reviewed article is published on protocols.io, https://dx.doi.org/10.17504/protocols.io.kxygx34jdg8j/v1, and is included for printing as an S1 File with this article. In this protocol, we offer two guides. The first details how to translate iatgen instructions, starting from either vetted, unvetted, or new translations, into various languages and through either a web interface or an R package. The second documents the procedures for conducting IATs in Qualtrics offline, for instance, in environments without internet connectivity.

### Expected results

The protocol provides instructions for using the translation features recently added to iatgen. The first three tutorials (*Web Interface – Using Vetted Translations*; *tr.iatgen Package in R – Using Vetted Translations*; and *Web & R – Converting an IAT to Another Language based on an Unvetted Translation*) product a (QSF) file which can be imported into Qualtrics creating an IAT with instructions in the desired language. The “*Web & R – Creating a New Translation*” tutorial provides the necessary steps for translating iatgen’s IAT instructions while preserving their graphical layout (e.g., maintaining bold and italic text rendering). The resulting spreadsheet file is compatible with both iatgen’s web app and the tr.iatgen package.The “*Building the GitHub Translation Repository*” protocol will grant access to the source files with existing translations and instructions for submitting new translations.

Following the “*Obtaining access to offline surveys with Qualtrics*” protocol instructs users on enabling the offline survey option in Qualtrics. Likewise, “*Fine-tuning iatgen for Offline Studies (When using Images)*” results in a Qualtrics file (QSF) optimized for offline data collection. Note that the fine-tuning tutorial is only necessary for offline IATs with images. Finally, “*Downloading and Conducting IATs Offline*” outlines the steps required to run an offline IAT with iatgen+Qualtrics’ mobile app.

### User testimonials and case studies

The following testimonials demonstrate the practical impact of iatgen’s new features (see supplementary material for the complete testimonials). These are not meant as empirical evidence, as iatgen has already been validated elsewhere ([8]). These testimonials are meant as evidence of tr.iatgen’s functionality and usability for researchers and as examples of relevant use cases.

Michelle Zandbergen at Erasmus Medical Center (Netherlands) used the tr.iatgen package to examine attitudes toward depression across ethnically diverse populations (Appendix A). Stigma toward mental illness often impedes access to care, so the goal of the study was to understand how attitudes toward mental and physical illnesses differ across demographic groups. Zandbergen collected data using an IAT and the Depression Stigma Scale (DSS) in five languages—Dutch, English, Turkish, Persian, and Arabic—at a B1 literacy level to ensure accessibility for individuals with varying educational attainment. “The flexibility of iatgen,” Zandbergen asserts, “simplified the customization of these multilingual IATs and accelerated data collection, ensuring that we could capture insights from a broad demographic.”

Gonçalo Freitas at Institute of Social Sciences – University of Lisbon (Portugal), conducting research in Portugal, investigated implicit national identification using the IAT (Appendix B). “The translation package developed by Santos and collaborators allowed me to run the experiment entirely in Portuguese,” Freitas notes. “The iatgen Shiny app’s intuitive design was key to streamlining the data analysis. Uploading the Qualtrics data and generating results took only a few seconds, saving considerable time and minimizing errors.” The accessibility and efficiency offered by the platform allowed Freitas to focus on the theoretical implications rather than technical complications.

Tailson Mariano at Universidade Católica de Pernambuco (Brazil), explored the influence of video game characters’ skin color on racial attitudes and aggression levels among players (Appendix C). “We aimed to determine whether the skin color of violent game characters affected the attitudes and behaviors of Black and White participants differently,” Mariano explains. “Using the translated version of iatgen was critical—it enabled us to create IATs tailored to our objectives, ensuring that our measures accurately captured implicit racial biases.” The platform’s user-friendly interface facilitated data collection and analysis, helping the research team navigate language challenges and generate meaningful insights into the interplay between race, media, and aggression.

Angél del Fresno Diáz at the University of Gdańsk (Poland), used iatgen to conduct an IAT study with a Spanish-speaking sample (Appendix D). Fresno-Diáz noted “the instructions to build the IAT for Qualtrics is a very simple process. You just need to follow the steps that are carefully described.” He further noted, however, that he experienced technical difficulties with string encoding when creating a new translation in Spanish. “I had some problems with symbols such as accents.” After reaching out to the authors, the issue was quickly resolved. “Still, you helped me to fix these issues very quickly! But it’s true that without your help I wouldn’t have managed to build my own template in Spanish.” Fresno-Diáz’s translation has since been added to the official list, making it accessible to others working with Spanish-speaking samples.

Do Bú and collaborators, conducting research in Brazil and Portugal, explored implicit racial biases among healthcare providers [42–44] (Appendix E). “The new iatgen features were instrumental for our study,” they report. “Given the time constraints of healthcare professionals, the ability to collect data remotely using a single link was invaluable. The dual-language support—enabling both Brazilian and European Portuguese—was crucial in engaging participants across both countries.” The functionality not only simplified recruitment but also facilitated data processing: “With a few clicks, we were able to analyze implicit bias scores, making the entire process remarkably efficient.”

Finally, Hara and collaborators are conducting a field survey in Nairobi, Kenya, targeting vulnerable youths in informal settlements (Appendix F). This study, aimed at understanding vulnerability and conflict across religious and ethnic groups, exemplifies the importance of iatgen’s offline capabilities. “The survey will be conducted in local community halls spreading multiple locations, with some areas lacking stable internet or mobile connection,” Hara explained. “In such cases, the offline functionality of iatgen, combined with Qualtrics, ensures seamless survey execution and streamlines data analysis.” This adaptability will enable the team to engage with populations who are often excluded from traditional research due to technological barriers.

These examples illustrate how iatgen’s new features empower researchers to overcome logistical challenges, expanding the application of IATs to more diverse populations.

## Conclusion

Iatgen has proven to be a useful tool for researchers in many fields. In this paper we introduce a new translation package – tr.iatgen – that allows users to create IATs in any language. Many validated IAT translations are easily accessible on the web-based iatgen Shiny app. We invite readers to contribute to the translation repository in GitHub by contributing and reviewing IAT translations. The translations in GitHub can be implemented (after accuracy verification) using the web-based iatgen shiny app or tr.iatgen package in R. We also describe the steps required to conduct iatgen IATs without internet access using the offline Qualtrics application.

The enhanced translation and offline features described here improve iatgen’s versatility and may help reduce the negative effects of WEIRD samples. Notably, in suitable use cases, iatgen simplifies time-consuming tasks such as IAT experiment programming, data collection, cleaning, and analysis. With offline and non-English translation features, iatgen can further free researchers from constraints based on participants’ English fluency or internet connectivity. This can allow scholars to focus on more important aspects of research such as generating interesting and meaningful hypotheses. We hope this work facilitates research with more diverse participant samples around the world.

## Supporting information

S1 File Protocol Description. (PDF)

S1 Appendix Testimonial 1. (PDF)

S2 Appendix Testimonial 2. (PDF)

S3 Appendix Testimonial 3. (PDF)

S4 Appendix Testimonial 4. (PDF)

S5 Appendix Testimonial 5. (DOCX)

S6 Appendix Testimonial 6. (DOCX)
