# Supplementary material for: Implicit association tests for all: Using iatgen for non-English and offline samples
Source: PLoS One. 2026 Apr 17;21(4):e0342742. doi: 10.1371/journal.pone.0342742 (PMC13089732; doi:10.1371/journal.pone.0342742)
Supplement: S1 File — (PDF) [file pone.0342742.s001.pdf]

Jan 03, 2024

Generating Non-English IATs and Collecting Offline Samples

DOI
https://dx.doi.org/10.17504/protocols.io.kxygx34jdg8j/v1

João Oliveira Santos[1], Emerson Araújo Do Bú[2], Tomohiro Hara[3], Cristina Mendonça[1], Sara Hagá[1], Ruth Pogacar[4], Michal Kouril[5,6]

[1]CICPSI, Faculdade de Psicologia, Universidade de Lisboa, Portugal;
[2]Department of Public Health Sciences, University of Virginia, Charlottesville, VA, USA;
[3]Musashi University, Tokyo, Japan; [4]University of Calgary, Calgary, Alberta, Canada;
[5]University of Cincinnati College of Medicine, Cincinnati, OH, USA;
[6]Cincinnati Children's Hospital Medical Center, Cincinnati, OH, USA

João Oliveira Santos: Department of Public Health Sciences, University of Virginia, Charlottesville, VA, USA;

João Oliveira Santos

Create & collaborate more with a free account

Edit and publish protocols, collaborate in communities, share insights through comments, and track progress with run records.

Create free account

OPEN ACCESS

DOI: https://dx.doi.org/10.17504/protocols.io.kxygx34jdg8j/v1

Protocol Citation: João Oliveira Santos, Emerson Araújo Do Bú, Tomohiro Hara, Cristina Mendonça, Sara Hagá, Ruth Pogacar, Michal Kouril 2024. Generating Non-English IATs and Collecting Offline Samples. **protocols.io**
https://dx.doi.org/10.17504/protocols.io.kxygx34jdg8j/v1

License: This is an open access protocol distributed under the terms of the **Creative Commons Attribution License**, which permits unrestricted use, distribution, and reproduction in any medium, provided the original author and source are credited

Protocol status: Working
We use this protocol and it's working

Created: December 24, 2023

Last Modified: January 03, 2024

Protocol Integer ID: 92708

Keywords: iats in offline environment, using iatgen, repository of translation, based iat, collecting offline sample, iatgen, translation, offline samples this protocol, iat, flexibility of the iat, offline environment, qualtric, sample, tool

Funders Acknowledgements:
Fundação para a Ciência e Tecnologia
Grant ID: PD/BD/135465/2017
Fundação para a Ciência e Tecnologia
Grant ID: NT-DL57-4652

Abstract

This protocol describes tools to: a) create non-English IATs using iatgen and R, b) build the repository of translations for iatgen, and c) deploy Qualtrics-based IATs in offline environments. Together, these functions increase the flexibility of the IAT and may help reduce the negative effects of WEIRD samples

Attachments

S1_file.pdf
188KB

Troubleshooting
