# Supplementary material for: Implicit association tests for all: Using iatgen for non-English and offline samples
Source: PLoS One. 2026 Apr 17;21(4):e0342742. doi: 10.1371/journal.pone.0342742 (PMC13089732; doi:10.1371/journal.pone.0342742)
Supplement: S1 Appendix — (PDF) [file pone.0342742.s002.pdf]

Erasmus MC
Universitair Medisch Centrum Rotterdam

Department of Public Health
Erasmus Medical Center
Dr. Molewaterplein 40
3015 GD Rotterdam, Netherlands

E: m.zandbergen@erasmusm.nl

Date: 01-10-2024
Subject: Testimonial on Translation Capabilities of tr.iatgen

Dear,

As a second-year PhD student in the Department of Public Health at Erasmus Medical Center, I will be investigating implicit and explicit attitudes towards depression in an ethnically diverse adult population. My research will address a significant gap in understanding how these attitudes can vary based on demographic factors such as ethnicity, age, gender, and educational background.

Depression is a prevalent mental health condition that affects millions globally and is often associated with stigma and negative perceptions. Understanding how these attitudes differ between mental and physical illnesses is crucial for improving mental healthcare access and reducing barriers to treatment. My study will employ both implicit association tests (IATs) and the Depression Stigma Scale (DSS) questionnaire to assess these attitudes, allowing for a comprehensive analysis of how individuals perceive depression compared to physical health conditions.

Given the diversity of the population we aim to study, a critical component of my research is its inclusivity. To ensure that language barriers do not exclude potential participants, I organized the IAT and DSS questionnaire in multiple languages, including Dutch, English, Turkish, Persian and Arabic. This linguistic diversity is essential for engaging a broader demographic, including individuals with varying educational backgrounds. To further enhance accessibility, I designed the assessments to be at a B1 literacy level, enabling participation from those with lower educational attainment.

Utilizing the tr.iatgen package to implement the IAT in different languages has been invaluable. Its user-friendly interface and comprehensive documentation have made it easy for someone with my background in Neurobiology, but limited programming experience, to

navigate and customize the IAT for my research needs. The ability to quickly adapt the tool to fit my study's requirements has greatly accelerated my research process.

Throughout this journey, I encountered some technical difficulties with the template form due to unexpected changes on my laptop. However, the prompt and supportive assistance from the tr.iatgen team was incredibly reassuring and helped me overcome these challenges in a short time period.

In conclusion, the tr.iatgen package has been instrumental in the successful execution of my research project. Its ease of use and adaptability have allowed me to focus on my study's primary objectives—advancing our understanding of attitudes towards depression and reducing stigma in diverse populations. I highly recommend tr.iatgen to fellow researchers in the field of public health and beyond.

Michelle Zandbergen
PhD Candidate
Department of Public Health
Erasmus Medical Center, Netherlands
