# Supplementary material for: Implicit association tests for all: Using iatgen for non-English and offline samples
Source: PLoS One. 2026 Apr 17;21(4):e0342742. doi: 10.1371/journal.pone.0342742 (PMC13089732; doi:10.1371/journal.pone.0342742)
Supplement: S2 Appendix — (PDF) [file pone.0342742.s003.pdf]

Appendix B

I conducted an experiment on implicit national identification using IAT with national-related stimuli. The translation package developed by Santos and collaborators made it easier for me to conduct the experiment as the native language of my sample was Portuguese. The shiny app developed by the team is intuitive and clear to use, having an incredible plus which is the possibility to upload the datafile exported from Qualtrics and have the data analyzed in a few seconds.

Gonçalo Freitas, Institute of Social Sciences, University of Lisbon, Portugal
