# Supplementary material for: Implicit association tests for all: Using iatgen for non-English and offline samples
Source: PLoS One. 2026 Apr 17;21(4):e0342742. doi: 10.1371/journal.pone.0342742 (PMC13089732; doi:10.1371/journal.pone.0342742)
Supplement: S3 Appendix — (PDF) [file pone.0342742.s004.pdf]

Appendix C

In our study, we aimed to examine the influence of video game characters' skin color on both implicit and explicit racial attitudes and aggression levels among participants. Specifically, our research sought to understand if the skin color of violent video game characters could affect the attitudes and behaviors of Black and White players differently. Using the translated version of iatgen was instrumental in this research. It allowed us to seamlessly create and translate Implicit Association Test (IAT) measures, making it easy to assess participants' implicit racial attitudes. The tool's flexibility and user-friendly interface facilitated the creation of tailored IATs that accurately reflected the study's objectives. Additionally, the ease of using iatgen helped streamline the experimental process, allowing us to quickly analyze if participants expressed different levels of aggression depending on the skin color of the character they played. This tool was critical in ensuring that we could collect reliable, valid data with minimal technical hurdles (e.g., language), enabling us to focus more on the theoretical implications of our findings. In conclusion, the translated version of iatgen was not only efficient but also greatly contributed to the quality and depth of our analysis, providing clear insights into the intersection of race, media, and aggression.

Tailson Mariano, Universidade Católica de Pernambuco, Brazil
