# Supplementary material for: Implicit association tests for all: Using iatgen for non-English and offline samples
Source: PLoS One. 2026 Apr 17;21(4):e0342742. doi: 10.1371/journal.pone.0342742 (PMC13089732; doi:10.1371/journal.pone.0342742)
Supplement: S4 Appendix — (PDF) [file pone.0342742.s005.pdf]

Appendix D

I think the blog where all the information is available, as well as the instructions to build the IAT for qualtrics is a very simple process. You just need to follow the steps that are carefully described. The main difficulty for me is if you want to include a new language that does not appear in the list of available languages. It is a process that at first look seems simple (filling in the template) but I had some problems with symbols such as accents. So I tried numerous times to make changes to the template to try to make it work, but it didn't work. Still, you helped me to fix these issues very quickly! But it's true that without your help I wouldn't have managed to build my own template in Spanish.

Angél del Fresno Diáz, University of Gdańsk, Poland
