# Supplementary material for: Implicit association tests for all: Using iatgen for non-English and offline samples
Source: PLoS One. 2026 Apr 17;21(4):e0342742. doi: 10.1371/journal.pone.0342742 (PMC13089732; doi:10.1371/journal.pone.0342742)
Supplement: S5 Appendix — (DOCX) [file pone.0342742.s006.docx]

**Appendix E**

In our recent study on implicit racial biases among healthcare providers in Brazil and Portugal, the new iatgen features were instrumental for our research. Given the time constraints of healthcare professionals, the ability to collect data remotely using a single link was invaluable. The dual-language support (enabling both Brazilian and European Portuguese) was crucial in engaging participants across both countries. This functionality not only simplified participant recruitment but also made data processing efficient, allowing us to analyze implicit bias scores with ease. Iatgen’s innovative features truly enhanced our research capacity and provided seamless support for our cross-country investigation.

**Emerson Do Bú, Institute of Social Sciences, University of Lisbon, Lisbon, Portugal**
