# Supplementary material for: Implicit association tests for all: Using iatgen for non-English and offline samples
Source: PLoS One. 2026 Apr 17;21(4):e0342742. doi: 10.1371/journal.pone.0342742 (PMC13089732; doi:10.1371/journal.pone.0342742)
Supplement: S6 Appendix — (DOCX) [file pone.0342742.s007.docx]

**Appendix F**

My collaborators and I are conducting a field survey in Nairobi, Kenya, targeting vulnerable youths in informal settlements. The survey will be conducted in local community halls spreading multiple locations, with some areas lacking stable internet or mobile connection. The survey will be conducted in Kiswahili, the local language, and IAT is just one part of a broader survey module aimed at understanding vulnerability and conflicting relationships across different ethnic/religious groups. In such a case, the offline feature of IAT, combined with Qualtrics in a local language, allows for seamless execution of the entire survey, and streamlines data analysis.

**Tomohiro Hara, Musashi University, Tokyo, Japan**
